# Detection of copy number variation associated with ventriculomegaly in fetuses using single nucleotide polymorphism arrays

**DOI:** 10.1038/s41598-021-83147-7

**Published:** 2021-03-05

**Authors:** Huili Xue, Aili Yu, Na Lin, Xuemei Chen, Min Lin, Yan Wang, Hailong Huang, Liangpu Xu

**Affiliations:** 1 Department of Fujian Key Laboratory for Prenatal Diagnosis and Birth Defect, Fujian Maternity and Child Health Hospital, Affiliated Hospital of Fujian Medical University, Gulou, Fuzhou, 350001 Fujian Province China; 2 Reproductive Medicine Center, Fujian Maternity and Child Health Hospital, Affiliated Hospital of Fujian Medical University, No. 18 Daoshan Road, Gulou District, Fuzhou City, 350001 Fujian Province China

**Keywords:** Genetics, Molecular medicine

## Abstract

Etiopathogenesis of fetal ventriculomegaly is poorly understood. Associations between fetal isolated ventriculomegaly and copy number variations (CNVs) have been previously described. We investigated the correlations between fetal ventriculomegaly—with or without other ultrasound anomalies—and chromosome abnormalities. 222 fetuses were divided into four groups: (I) 103 (46.4%) cases with isolated ventriculomegaly, (II) 41 (18.5%) cases accompanied by soft markers, (III) 33 (14.9%) cases complicated with central nervous system (CNS) anomalies, and (IV) 45 (20.3%) cases with accompanying anomalies. Karyotyping and single nucleotide polymorphism (SNP) array were used in parallel. Karyotype abnormalities were identified in 15/222 (6.8%) cases. Karyotype abnormalities in group I, II, III, and IV were 4/103 (3.9%), 2/41 (4.9%), 4/33 (12.1%), and 5/45 (11.1%), respectively. Concerning the SNP array analysis results, 31/222 (14.0%) were CNVs, CNVs in groups I, II, III, and IV were 11/103 (10.7%), 6/41 (14.6%), 9/33 (27.3%), and 5/45 fetuses (11.1%), respectively. Detections of clinical significant CNVs were higher in non-isolated ventriculomegaly than in isolated ventriculomegaly (16.81% vs 10.7%, P = 0.19). SNP arrays can effectively identify CNVs in fetuses with ventriculomegaly and increase the abnormal chromosomal detection rate by approximately 7.2%, especially ventriculomegaly accompanied by CNS anomalies.

## Introduction

Fetal lateral ventricle width is a common test parameter in prenatal ultrasound screening. At 15 to 40 weeks of gestation, the lateral ventricular width is normally 7.6 ± 0.6 mm. A width of one or both lateral ventricles between 10.0 and 15.0 mm indicates mild ventriculomegaly (MVM). Widths > 15.0 mm indicate severe ventriculomegaly (SVM). When ventriculomegaly (VM) is detected, level III ultrasound scanning and fetal brain magnetic resonance imaging are performed to identify the progression of VM and detect the presence of intracranial and extracranial structural abnormalities.

Fetal VM is one of the most common indicators of central nervous system (CNS) abnormalities in prenatal ultrasound screening. VM is a nonspecific soft marker of prenatal ultrasound screening and is the most common hallmark of fetal CNS abnormalities. Fetal VM has been associated with autism, schizophrenia, epilepsy, and other neurodevelopmental disorders (NDDs)^1^, affecting 1% of fetuses and is associated with cognitive, language, and behavioral disorders in children^2^. The causes of VM are complex and include obstruction of the cerebrospinal fluid flow and focal or generalized brain volume loss, and are secondary to the CNS deformity^3^. The prognosis of affected fetuses is influenced by the chromosome abnormalities, severity of VM, viral infection, presence of structural anomalies in other systems, and evolution in utero. The incidence of chromosomal abnormalities in fetuses with VM varies from 5 to 8.3%^4^. Hence, chromosomal abnormality is an important cause of VM.

Routine karyotype analysis is a common method for detecting chromosomal abnormalities and plays an important role in the diagnosis of fetal VM. However, it is difficult to detect copy number variations (CNVs) owing to limited resolution. Chromosomal microarray analysis has improved the detection rate of CNVs that cannot be diagnosed using karyotyping. With widespread implementation of SNP array, more pathogenic CNVs have been prenatally detected^5^. SNP array technology is recommended as a routine, first tier procedure for almost all prenatal diagnoses. CNVs have been reported in approximately 6% of fetuses with ultrasound anomalies (UAs) and a normal karyotype^6^. SNP array can increase the rate of diagnosis by 12–15% in cases with structural malformations^7,8^.

SNP array has revealed additional pathogenic causes of NDDs and craniofacial malformations^9^. The risk of chromosomal abnormalities for fetuses with isolated ventriculomegaly (IVM) is high, especially when SVM (which is bilateral and progressive) occurs in the second trimester^10^. Gezer et al.^11^ reported an 8.6% rate of chromosomal abnormalities in fetuses with IVM. Hu et al.^12^ reported an 8.4% rate of abnormal CNVs in fetuses with IVM. Few studies have focused on CNVs findings in fetuses with VM accompanied with other UAs. In our cohort, 222 pregnant women with VM fetuses consented to SNP array and karyotyping in parallel. We retrospectively analyzed the prenatal diagnosis results of these 222 fetuses and clarified the association between fetal VM with or without other UAs and chromosomal abnormalities.

## Methods

### Ethical statement

This study was reviewed and approved by the Ethics Review Committee of Fujian Maternity and Child Health Hospital (approval no.: 2007-0112). Signed informed consent was obtained from all participants or parents following a detailed description of the purpose of the study. All experiments performed involving human participants were in accordance with the ethical standards of the institutional and/or national research committee and with Helsinki declaration and relevant guidelines and regulations.

### Patients’ data

This retrospective study involved cases of fetal VM diagnosed using prenatal ultrasound between July 2015 and June 2019 in Fujian Maternity and Child Health Hospital. All the pregnant women provided written informed consent as disclosed in the Ethics Declaration above. Fetal cell samples were collected by amniocentesis between 16 and 22 weeks of gestation or by cordocentesis after 22 weeks of gestation. The cases were divided into four groups. Group I comprised cases of IVM (n = 103). Group II comprised VM in association with soft markers (n = 41). Group III comprised VM complicated with CNS anomalies (n = 33). Group IV comprised VM accompanied by other UAs (n = 45). Obstetric outcomes were followed up from January to August 2020. Developmental data of the infants born were collected and assessed postnatally based on medical documents or telephone conversations at median infant age of 2 years.

### Conventional chromosomal karyotyping

Routine cytogenetic analyses at a resolution of approximately 400 to 500 bands were performed according to standard laboratory protocols. 20 mL of amniotic fluid were subjected to in situ amniocyte culture, harvesting, and G-banding. Parental blood samples were also collected for cytogenetic analyses, when necessary. Fifteen primary colonies were examined by standard analysis. Where fewer than 15 primary colonies were available, 20 cells from both primary and trypsinized cultures were examined.

### Single nucleotide polymorphism (SNP) array

Whole genomic DNA was extracted from amniocytes or fetal cord blood of each fetus and its parents using the QIAamp DNA Blood Mini Kit (Qiagen, Valencia, CA). The DNA was examined by SNP array analysis using the CytoScan 750 K Array (Affymetrix, Santa Clara, CA) containing 200,000 SNPs and 550,000 CNVs as previously described^13^. The results were analyzed using the Chromosome Analysis Suite software (Affymetrix) and annotated based on GRCh37 (hg19). The detected CNVs were compared to the Database of Genomic Variants (DGV), DECIPHER, Online Mendelian Inheritance in Man (OMIM), International Standards for Cytogenomic Arrays, University of California Santa Cruz, PubMed, and the CliGen Resource. The reporting threshold was set at gains or losses ≥ 400 Kb and loss of heterozygosity ≥ 5 Mb. The CNV reporting filter was set at > 200 Kb with a minimum set of 50 marker counts and the SNP reporting filter was set at > 50 Kb with a minimum set of 25 marker counts. For VM fetuses with abnormal CNVs, parental testing was performed to determine the inheritance pattern of the abnormal CNVs. All nucleotide positions of CNVs refer to the Human Genome Feb 2009 Assembly (GRCh37/hg19). The classification of CNVs is performed referring to Fig. 4. The CNVs were validated using fluorescence in situ hybridization or quantitative fluorescent polymerase chain reaction.

### Statistical analysis

Statistical analysis was performed using SPSS software v19.0 (SPSS Inc, Chicago, IL). Comparisons were performed using *two-tailed* test and *chi-square* test on the independent samples. A *p*-value < 0.05 was considered statistically significant.

## Results

### Patient clinical characteristics

**Figure 1:**
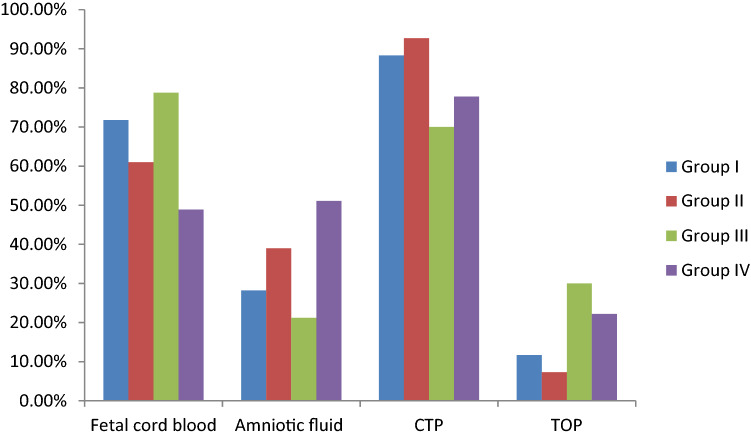
Demographic characters except for gestation age at invasive prenatal diagnosis for 222 pregnant women of ventriculomegaly in fetuses.

**Figure 2:**
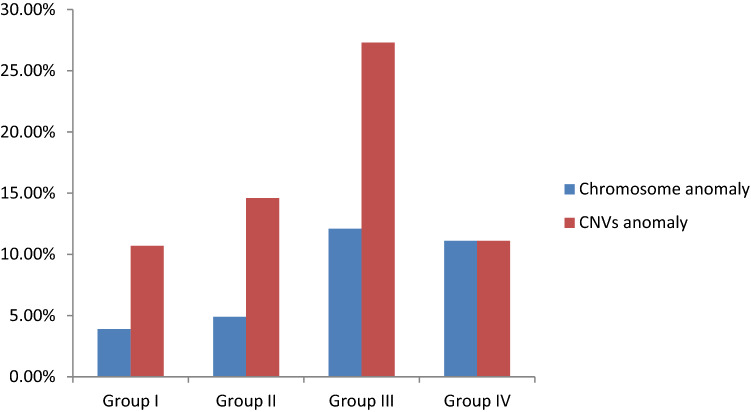
The chromosome and CNVs anomaly among four groups.

**Figure 3:**
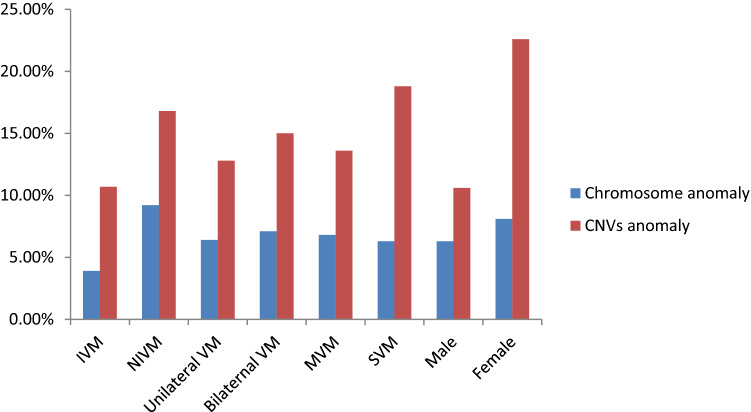
Classification characteristics of 222 fetuses with ventriculomegaly.

A total of 222 prenatal cases were enrolled in this study. The demographic characteristics, prenatal ultrasound findings, karyotypes, SNP array results, pregnancy outcomes, and available postnatal findings of 33 cases are summarized in Tables 1, 2, 3 and Figs. 1, 2, 3. The 222 cases were allocated to group I (n = 103, 46.4%), II (n = 41, 18.5%), III (n = 33, 14.9%), and IV (n = 45, 20.3%), as describes earlier.

**Table 1:** Demographic characters for 222 pregnant women of ventriculomegaly in fetuses.

	All (n = 222)	Group I (n = 103)	Group II (n = 41)	Group III (n = 33)	Group IV (n = 45)
**Gestation age at invasive PD (wk):mean ± SD**	21.4 ± 3.2	23.1 ± 2.3	22.4 ± 1.4	24.6 ± 2.8	25.0 ± 2.5
**Specimens**					
Cord blood n (%)	147 (66.2%)	74 (71.8%)	25 (61.0%)	26 (78.8%)	22 (48.9%)
Amniotic fluid n (%)	75 (33.8%)	29 (28.2%)	16 (39.0%)	7 (21.2%)	23 (51.1%)
**Pregnancy outcomes**					
CTP n (%)	187(84.7%)	91 (88.3%)	38 (92.7%)	23(72.7%)	35(77.8%)
TOP n (%)	34(15.3%)	12(11.7%)	3(7.3%)	10(27.3%)	10(22.2%)

*PD* prenatal diagnosis, *SD* standard deviation, *CTP* continuation of pregnancy, *TOP* termination of pregnancy.

**Table 2:** Classification characteristics of 222 fetuses with ventriculomegaly.

VM classification	Number of fetuses with anomaly (%total cohort)	Number of fetuses with chromosome anomaly (%total cohort)	Number of fetuses with CNV (%total cohort)
Severity of VM	222 (100%)		
Mild	206 (92.8)	14(6.8%)	28 (13.5%)
Severe	16 (7.2%)	1(6.7%)	3 (20%)
VM with fetus gender	222 (100%)		
Male	160(72.1)	10(6.3%)	17 (10.6%)
Female	62(27.9%)	5(8.1%)	14 (22.6%)
Laterality of VM	222 (100%)		
Unilateral	109(49.1)	5(4.6%)	14 (5.5%)
Bilateral	113(50.9%)	8(7.1%)	17 (8.0%)

*VM* ventriculomegaly, *CNV* copy number variation.

**Table 3:** Details of the fetuses with clinical significant CMA findings in Group I, II, III and IV.

Case number	Ultrasound findings	SNP array results	Karyotype results	Parental testing	Associated syndrome	Pregnancy outcome
**Group I **						
Microscopic						
1	Ventriculomegaly	arr[GRCh37] (21)×3	47,XY,+21	dn	DS	TOP
Submicroscopic						
2	Ventriculomegaly	arr[GRCh37] 16p11.2 (29567296_30190029)x1	46,XX	dn	16p11.2 microdeletion syndrome	TOP
3	Ventriculomegaly	arr[GRCh37] 16p11.2 (29591326_30176508)x1	46,XY	dn	16p11.2 microdeletion syndrome	TOP
4	Ventriculomegaly	arr[GRCh37] 14q21.2q21.3(46782405_49288860)x1	46,XX	NA	Non-syndromic	TOP
5	Ventriculomegaly	arr[GRCh37] 3p22.1 (42875130_43309436)x1	46,XY	NA	Non-syndromic	TOP
6	Ventriculomegaly	arr[GRCh37]Xq28(152446333_153581657)x3, 1p36.33p36.23(849466_8592172)x1, 1q44(246015892_249224684)x3	46,XX,t(5;14) (p13.3;q21)	Mat	MECP2 duplication syndrome 1p36 microdeletion syndrome Non-syndromic	TOP
7	Ventriculomegaly	arr[GRCh37]3p26.3 (1855754_2663625)x1	46,XX	NA	Non-syndromic	CTP
8	Ventriculomegaly	arr[GRCh37]16p13.11 (15058820_6309046)x3	46,XX	Pat	16p13.11 microduplication syndrome	TOP
9	Ventriculomegaly	arr[GRCh37] 7q36.3(155347675_156348660)x3	46,XY	dn	Non-syndromic	TOP
10	Ventriculomegaly	arr[GRCh37]15q11.2q13.1(23290787_28540345)x1	46,XX	NA	AS/PWS	TOP
11	Ventriculomegaly	arr[GRCh37] 7q36.3 (158875328_159119707)x1, 9q34.11q34.3 (132845830_141018648)x3	46,XY	NA NA	Non-syndromic Duplication 9q34 syndrome	Live birth
**Group II**						
Microscopic						
12	Bilateral ventriculomegaly mild tricuspid regurgitation	arr[GRCh37]21q11.2q22.3 (15016486_48093361)x3	47,XY,+21	NA	DS	TOP
13	Bilateral ventriculomegaly NT thichness	arr[GRCh37] (21)x3	47,XY,+21	NA	DS	TOP
Submicroscopic						
14	Left lateral ventriculomegaly mild tricuspid regurgitation	arr[GRCh37]18q11.2 (19620590_21572153)x3	46,XY	Pat	Non-syndromic	TOP
15	Bilateral ventriculomegaly echogenic bowel	arr[GRCh37]16p13.11 (15422960_16508123)x1	46,XY	dn	16p13.11 microduplication syndrome	TOP
16	Left lateral ventriculomegaly echogenic bowel ventricular echogenicity	arr[GRCh37]16p11.2 (28810324_29032280)x1	46,XY	NA	16p11.2 microdeletion syndrome	TOP
17	Left lateral ventriculomegaly echogenic bowel ventricular echogenicity	arr[GRCh37]15q13.3 (32021609_32444043)x3	46,XY	NA	15q13 microduplication syndrome	CTP
**Group III**						
Microscopic						
18	Bilateral ventriculomegaly absence of CSP	arr[GRCh37]21q11.2q22.3 (15016486_48093361)x3	47,XX,+21	NA	DS	TOP
19	Bilateral ventriculomegaly asymmetrically, ACC	arr[GRCh37]Xp22.33 (168551_2716151)x3 or Yp11.32(10001_2649520)x3, Yp11.31q11.221(2650424_18450717)x2, Yq11.221q11.23(18481644_28799654)x0	46,X,+mar[59]/45,X[6]	NA	Aazoospermia	TOP
20	Bilateral ventriculomegaly cerebellar hypoplasia prenasal soft tissue thickness	arr[GRCh37]5p15.33p13.3 (113576_32785953)x1, 9p24.3p21.3(208454_21354180)x3	46,XY,der(5) t(5;9) (p13.3;p21.3)mat	dn	CDCS	TOP
Submicroscopic						
21	Bilateral ventriculomegaly ACC	arr[GRCh37]1q21.1 (145375770_145770627)x1, 9p24.1(4623660-5501699)x3	46,XY,inv(9) (p12q13)	dn	1q21.1 diatal deletion syndrome Duplication 9p syndrome	TOP
22	Bilateral ventriculomegaly widening of the third ventricle largen CSP high risk for DS polyhydramnios	arr[GRCh37]5q35.2q35.3 (175416095_177482506)x1	46,XY	NA	Sotos syndrome	TOP
23	Bilateral ventriculomegaly bilateral choroid plexus cyst	arr[GRCh37]1q31.1 (186148297_190257668)x3	46,XX	Pat	Non-syndromic	Live birth
24	Right unilateral VM right choroid plexus cyst	arr[GRCh37]15q11.2 (22770421_23625785)x1	46,XY	Pat	15q11.2 deletion syndrome	Live birth
25	Left unilateral VM increased cerebellar cistern width ependymal cyst interpodal cyst	arr[GRCh37]11p15.1p14.3 (20745930_21780075)x3	46,XY	NA	Non-syndromic	TOP
26	Bilateral VM enhanced bilateral periventricular white matter ecnogenic CHD	arr[GRCh37]3q24q25.1 (143476996_151222561)x1	46,XX	NA	Wisconsin syndrome	Live birth
**Group IV**						
Microscopic						
27	Left unilateral ventriculomegaly AMA, VSD, FGR persistent left superior vena cava tricuspid regurgitation gallbladder enlargement	arr[GRCh37]16p13.3 (85880_536631)x1, 17q24.2q25.3 (64966574_81041823)x3	46,XY,add (16)(p13.3)	NA	ATR-16 syndrome Partial trisomy 17q	TOP
28	Hydrocephalus cleft lip and palate nasal bone dysplasia Echogenic bowel	arr[GRCh37](1-22)×2,(XN)×1	46,XY,t(10;17) (q26;p11.2)	NA	–	TOP
29	Bilateral ventriculomegaly VSD	arr[GRCh37]21q11.2q22.11(15478958_34591567)x1, 21q22.3 (45812741_46655785)x1, 21q22.3(46822918_47532860)x1	46,XY,-21,+ mar	NA	21q22.1 microdeletion syndrome	TOP
30	Left unilateral ventriculomegaly VSD	arr[GRCh37]4q25q28.1 (112192577_127874789)x1	46,XY,del(4) (q25q28)	NA	Rieger syndrome	TOP
31	Left unilateral ventriculomegaly VSD	arr[GRCh37](1-22)×2,(XN)×1	46,X,inv(Y)	Pat	–	Live birth
Submicroscopic						
32	Left unilateral ventriculomegaly muscular VSD thickened nuchal fold persistent left superior vena cava enlarged right atrium and right ventricle right subclavian artery vagus Blake 's cyst	arr[GRCh37]2q13 (111397196_113111856)x1	46,XX	Mat	2q13 microdeletion syndrom	TOP
33	Left unilateral ventriculomegaly fetal is greater than the GW	arr[GRCh37] 3q26.1q29(163256369_197791601) hmz, 5p13.1p11(41029137_46313469) hmz, 6q24.2q26(143341406_161527784) hmz, 12q13.2q21.2(56011100_77134151) hmz, 17q21.2q21.32(39639602_45479706) hmz, 21q21.3q22.2(28124165_42352287) hmz	46,XY	NA	LOH	CTP

*CTP* continuation of pregnancy, *TOP* termination of pregnancy, *DS* down syndrome, *Dn* de novo, *AS* angelman syndrome, *PWS* prader-willi syndrome, *SNP* single nucleotide polymorphism, *CNV* copy number variation, *NT* nuchal translucency, *Pat* paternal, *Mat* maternal, *CSP* cavum septum pellucidum, *ACC* agenesis of the corpus callosum, *CDCS* Cri-du-chat syndrome, *VM* ventriculomegaly, *VSD* ventricular septal defect, *FGR* fetal growth restriction, *LOH* loss of heterozygosity, *AMA* advanced maternal age, *GW* gestational week, *ATR-16* alpha-thalassemia retardation-16 syndrome, *NA* not available

^$^For case 7, at present, the child is 1.5 years old, and she is developing normally.

^&^For case 23, Brain MR was reexamined 1 month after birth, and the width of bilateral ventricles was 1.14 and 1.15 cm, respectively, and the child is two years old, and she is developing normally currently.

Parents of 12 fetuses were genotyped by SNP array to determine the origin of the CNVs. Among those, six were de novo variants, two were maternally, and four were paternally inherited. Among the fetuses with pathogenic/likely pathogenic CNVs, parental testing was carried out in five; two occurred de novo, and three cases were inherited from normal parents.

Of the 222 samples, Cord blood was the predominant specimen in groups I, II, and III, whereas the proportion of amniotic fluid in group IV was slightly higher, significant differences were found between groups I and IV (*p* = 0.007) as well as between groups III and IV (*p* = 0.007) in type of specimens; Couples continuing the pregnancy accounted for 84.7%, while 15.3% terminated pregnancy; Similarly, significant differences were observed between groups I and III (*p* = 0.01) as well as between groups II and III **(p** = 0.01**)** with respect to the pregnancy outcomes. Conversely, no significant differences were found among four groups in gestation age at the time of invasive prenatal testing (Fig. 1).

Regarding the severity of VM, 206 fetuses displayed MVM and 16 fetuses displayed SVM. There was no significant difference in the karyotype abnormality rates between the two groups (*p* > 0.05). There were 160 male fetuses and 62 female fetuses, respectively, with a significant sex-related difference in the karyotype abnormality rate (*p* = 0.02; Table 2). Unilateral and bilateral VM was present in 109 and 113 fetuses, respectively, with no significant difference in karyotype abnormality rate between the two groups (*p* > 0.05).

### Detection rates of abnormal karyotypes

Among the 222 fetuses, 207 (93.2%) had normal karyotypes and 15 (6.8%) had abnormal karyotypes. Of the 15 cases, trisomy 21 was the most common aneuploidy, especially in group II. Karyotype abnormalities were detected in 4/103 cases (3.9%) in group I, 2/41 (4.9%) in group II, 4/33 (12.1%) in group III, and 5/45 (8.9%) in group IV. The frequency of clinically significant findings in group III was higher than that in groups I and II, but there was no significant difference among the four groups (*p* > 0.05) (Fig. 2).

The detection rate of karyotype abnormalities in fetuses with MVM and SVM was 14/206 (6.8%) and 1/16 (6.7%), respectively, with no significant difference (*p* > 0.05). The detection rate of karyotype abnormalities in male and female fetuses with VM was 10/160 (6.3%) and 5/62 (8.1%), respectively (*p* > 0.05). The detection rate of karyotype abnormalities in fetuses with IVM and non-IVM (NIVM) was 4/103 (3.9%) and 11/119 (9.21%), respectively (*p* > 0.05). The detection rate of karyotype abnormalities in fetuses with unilateral VM and bilateral VM was 7/109 (6.4%) and 8/113 (7.1%), respectively (*p* > 0.05).

### Detection rates of the clinical significant CNVs using SNP array

The rate of CNVs detected by SNP array in group I, II, III, and IV was 11/103 (10.7%), 6/41 (14.6%), 9/33 (27.3%), and 5/45 (8.9%), respectively (Fig. 2). Abnormal SNP array findings were detected in 31 (14.0%) fetuses in total. Among these cases, seven were consistent with the karyotype results, and 24 (10.8%) cases carry (likely) pathogenic CNVs ranging in size from 220 kb to 21 Mb. In addition, there were six (2.7%) cases of variants of unknown significance (VOUS).

Twenty-four (likely) pathogenic CNVs results included trisomy 21; microdeletions at 16p11.2, 1p36.33p36.23, 15q11.2q13.1, 15q11.2, 16p13.11, Yq11.221q11.23, 5p15.33p13.3, 1q21.1, and 5q35.2q35.3; and microduplications at Xq28, 16p13.11, 7q36.3, 9q34.11, 15q13.3, 9p24.3p21.3, and 11p15.1p14.3 (Table3). Pathogenic/likely pathogenic CNVs results were found in 18 cord blood and six amniotic fluid specimens.

**Figure 4:**
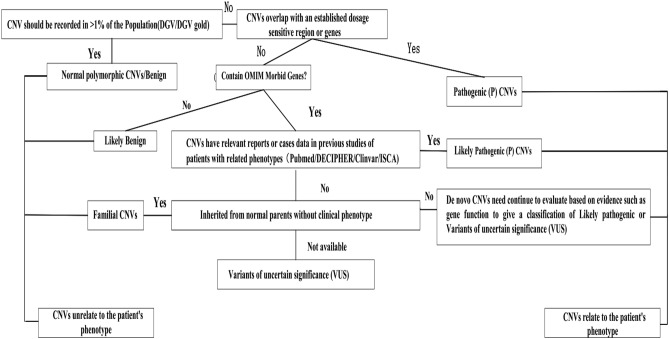
The flow chart of CNVs classification.

Across all groups, the highest frequency of CNVs was identified in group III (27.35%) followed by group II (14.6%). The difference in abnormal CNVs detection rates between groups I and III was statistically significant (*p* = 0.039). The difference in the detection rate of pathogenic / likely pathogenic CNVs between groups I and III was also significant (*p* = 0.0252). No significant differences were evident between the other groups. Of the 222 fetuses, there were 103 (46.4%) cases of IVM and 119 (53.6%) cases of NIVM. The detection rates of abnormal CNVs in fetuses with IVM and NIVM were 28/206 (13.6%) and 3/16 (18.8%), respectively (*p* > 0.05), 28/206 (13.5%), and 3/16 (20%) in fetuses with MVM and SVM, respectively (*p* > 0.05). The abnormal CNVs detection rates in male and female fetuses with VM was 17/160 (10.6%) and 14/62 (22.6%), respectively (p = 0.021) (Table 2). CNVs were also found in six (5.5%) fetuses with unilateral VM (109, 49.1%), and nine (8.0%) of the fetuses with bilateral VM (113, 50.9%) (Figs. 3, 4.).

### Detection rates of abnormal karyotypes and CNVs

The detection rates of abnormal karyotypes and CNVs were statistically significant (*p* = 0.017). Similarly, these rates of detection were significant for fetuses with IVM **(p** = 0.017), female fetuses (*p* = 0.025), and fetuses with bilateral VM (*p* = 0.031). There was no significant difference between abnormal karyotype and CNVs detection rates (*p* > 0.05) according to stratification based on the other types of VM.

### CNVs of unknown significance detected by SNP array in fetal VM with normal karyotypes

We detected six CNVs of unknown significance (VOUS). An atypical 2.5 Mb microdeletion at 14q21.2q21.3 was identified in case 4, which contained five genes: *LINC00871*, *RPL10L*, *MDGA2*, *MIR548Y*, and *LINC00648*. *MDGA2* is involved in the development of the nervous system. No prior report has described the pathologies related to this gene. An 808 Kb microdeletion at 3p26.3, was detected in case 7. The deletion included the *CNTN4* gene, which is associated with NDDs^14^. However, the behavioral influence of *CNTN4*, is unknown^15^. Other VOUS included losses of 434 Kb at 3p22.1 and 244 Kb at 7q36.3, and paternally inherited gains of 1.9 Mb at 18q11.2 and 4.1 Mb gain at 1q31.1. Three couples refused further genetic testing. Of these, only two CNVs were inherited from unaffected father and one occurred de novo. Four couples opted to terminate the pregnancy, and two couples continued the pregnancy. Labor was induced for one fetus with VOUS due to hydrocephalus.

### Clinical follow-up

Among the 24 fetuses with pathogenic / likely pathogenic CNVs, four were successfully delivered, nineteen were aborted, and one was unable to be located for follow-up. Among the four deliveries with pathogenic/likely pathogenic CNVs, three infants manifested NDDs, the remaining subject showed normal development. Among the three, two presented with motor delay and impaired language development, and one with global developmental delay (DD). Of 198 fetuses that did not harbor pathogenic CNVs, 186 showed normal development and 10 were lost to follow-up and labor was induced for two due to hydrocephalus and congenital heart disease, respectively.

## Discussion

MVM is an ultrasound soft marker, its detection indicates an increased risk of fetal chromosomal aneuploidy abnormalities, especially trisomy 21^16^. In our cohort, the CNVs detection rate of 14.0% was comparable to that reported in a previous study^17^, and the 10.7% rate of chromosome abnormalities in IVM was slightly higher than the previously reported rates of 7.8 to 8.4%^5,11,12^. The CNVs detected will enrich the data concerning CNVs of fetal VM. Pilu et al.^18^ reported a 3.8% rate of mean chromosomal abnormalities in fetuses with isolated MVM (IMVM). The differences between the studies could be due to differences in sample size, cohort sample selection, and array platforms. There was a significant difference in specimens types between groups I and IV (*p* = 0.007) as well as between groups III and IV (*p* = 0.007). These findings were closely related to the small sample size and to the fact that fetal craniocerebral anomalies are often diagnosed near or in the third trimester. Ultimately, cordocentesis may be necessary.

Compared with cordocentesis, amniocentesis is more popular due to the lower risk of fetal loss and the earlier gestation week. Considering the normal molecular cytogenetic results and slight UA, most pregnant women opted to continue their pregnancy. Pregnancy was terminated in case 15, considering the pathogenic CNVs. The increased diagnostic yield of SNP array for fetuses with UA, ranging from 5.2 to 10%, has been reported earlier^19^. An improved diagnostic yield of 7.2% by SNP array over karyotyping was observed in our cohort.

Although environmental factors—such as cytomegalovirus infection during the first trimester—can cause VM, genetic factors also play an important role. In our cohort, the overall detection rate of CNVs detected by SNP array was 14.0% (31/222). The detection rates of CNVs detected by SNP array in groups I, II, and III were significantly higher than the rate of karyotype abnormalities. Mercier et al.^20^ noted that the rate of chromosomal abnormalities in fetuses with IVM was higher than in fetuses with other UA, although not statistically significant. Gezer et al.^11^ reported the ultrasound soft marker group displayed the highest incidence of chromosomal abnormalities (10.5%), but the difference was not significant compared with other groups. Conversely, in our cohort, the highest frequency of pathogenic or likely pathogenic CNVs was detected in group III (27.3%), followed by group II (14.6%). Our findings support the recommendation for SNP array testing in cases of fetal VM, especially when accompanied by CNS anomalies. Nine fetuses harbored pathogenic CNVs in group III, of which 9p duplication syndrome was most frequently detected. The 9p duplication syndrome is characterized by dysmorphic craniofacial features, mental retardation, limb abnormalities, and short stature^21^. For fetus 20, SNP array analysis revealed a 32 Mb deletion at 5p15.33p13.3 (involving *CTNND2* and *DOCK8*) which caused cri-du-chat syndrome, and a 21 Mb duplication at 9p24.3p21.3 (involving *KANK1*) leading to 9p duplication syndrome. *CTNND2* is associated with reading disabilities and mild mild intellectual disabilities (ID)^22^. *CTNND2* haploinsufficiency contributes to cognitive dysfunction due to defective migration of neuronal cell subpopulations. These CNVs probably underlie multiple congenital anomalies. Thus, ruling out 9p duplication syndrome is recommended when fetal VM is accompanied by CNS anomalies.

In case 22, the fetus presented with bilateral VM, widening of the third ventricle and cavum septum pellucidum, polyhydramnios, and a high risk for Down syndrome. SNP array analysis in this case revealed a 2.0 Mb microdeletion in 5q35.2q35.3, encompassing 49 genes, including *NSD1*, which is associated with Sotos syndrome (SS). SS is characterized by excessively rapid growth, acromegalic features, non-progressive cerebral disorder, and NDDs^23^. The *NSD1* variant is responsible for approximately 90% of SS cases^24^. Furthermore, deletion of the *NSD1* gene is the most common cause of SS in the Japanese population^25^. We classified the CNV as a pathogenic variation, and parental SNP array testing was refused in this case.

A correlation between Wisconsin syndrome (WS) and 3q23q25 deletion has been described previously^26^. Bertini et al.^27^ reported a patient with a 6.5 Mb de novo deletion in 3q24q25.2. The patient presented with mild DD, absence of language acquisition, facial dysmorphism, hirsutism, strabismus, and dandy-walker malformation. In fetus 26, we identified a de novo 7.7 Mb deletion at 3q24q25.1, causing WS. The deletion encompassed 29 OMIM genes, including *ZIC1*, *ZIC4*, *MBNL2*, *ZCX1/2*, and *FOXL*. The deletion of *MBNL2*, *ZIC1*, and *ZIC4* correlated with cerebral anomalies. Thus, we classified the deletion at 3q24q25.1, as a pathogenic CNV. Although the fetus in case 26 presented with bilateral VM, enhanced bilateral periventricular, echnogenic white matter, and congenital heart disease. Parental SNP array testing was refused, and the couple opted to continue the pregnancy to term delivery. This infant was followe up to 4 years of age, and presented with moderate DD, speech delay, facial dysmorphism, and ventricular septal defect.

Our results also recommend the use of SNP array for fetuses with IVM, males, as well as bilateral VM. The detection rates of abnormal karyotypes and CNVs were unrelated to the other types and degree of VM. In addition, the average length of the lateral ventricle in fetuses with pathogenic/likely pathogenic CNVs was slightly smaller than that in fetuses without pathogenic/likely pathogenic CNVs. This finding may reflect the small sample size of our study. Alternately, it may be that severe fetal VM—such as hydrocephalus, whose cause is unknown—as further genetic testing was refused. Studies with larger number of patients are needed to provide clarity.

VM is more common in male fetuses^28^. The ratio in our cohort of male-to-female fetuses was 2.58:1, consistent with the values reported in the literature. Furthermore, the difference in the detection rate of CNVs was statistically significant between male and female fetuses (*p* = 0.021). However, the risk of abnormal karyotype in male fetuses was not. This reflects the higher resolution of SNP array technology compared to traditional karyotype analysis. SNP array can detect microdeletion/microduplications variants < 50–100 Kb in size.

The incidence of neurodevelopmental delay is 7.9–12% in IMVM fetuses^29^. IMVM has been correlated with NDDs, including autism spectrum disorder (ASD), attention deficit disorder, and learning disability in childhood as well as fetal or neonatal death^30^. Interestingly, the CNVs with clinically significant findings in groups I, II, and III were mostly associated with NDDs. Hence, SNP array increases the detection rate of pathogenic CNVs and reveals susceptibility loci (SL) for NDDs, which are often inherited from normal parents and lack phenotypic specificity. SL are generally associated with NDDs such as ASD, learning and speech problems, and varying degrees of intellectual disability or DD. Whether there is a causal relationship between SL for NDD and VM-related NDD remains to be confirmed.

Additionally, the specific molecular mechanism of SL that leads to VM-related NDD is unclear and requires further study. SL is considered the most common finding in pregnancies with a high risk of trisomy 21, 18, and 13^31^. CNVs associated with SL for NDDs are found in 1 to 3% of fetuses^32^. In our study, 3.6% (8/222) of fetuses harbored SL. The eight cases included 16p11.2 proximal deletion, with variations in the *TBX6 gene* (cases 2 and 3); 16p13.11 duplication, involving *NDE1* and *MYH11* genes (cases 8 and 15); 16p11.2 distal deletion, involving *SH2B1* (case 16); 15q13 duplication, involving *CHRNA7* (case 17); 1q21.1 BP3-BP4 distal deletion, involving *GJA5* (case 21); and 15q11.2 distal deletion, involving *NIPA1* (case 24). Of the fetuses with SL, there were three cases of low penetrance (< 10%; cases 8, 15, and 17), two cases of moderate penetrance (cases 21 and 24), and three cases with high penetrance (> 40%; cases 2, 3, and 16).

For fetus 21, SNP array analysis revealed a 395 Kb distal deletion in 1q21.1 (BP3-BP4), including *GJA5*. The estimated penetrance was 36.9%^33^, leading to the 1q21.1 distal deletion syndrome associated with GDD, ID, microcephaly, hypotonia, cardiac defects, and other neuropsychiatric disorders. *GJA5* is a candidate gene for ventricular septal defect ^34^. The gene encodes the cardiac gap junction protein connexin 40. It is well known that variations occurring de novo are more pathogenic, and inherited aberrations are more benign. However, CNVs such as the 1q21.1 distal microdeletion are especially important. Even if inherited from normal parents, both affected individuals and healthy controls can be pathogenic^35^. The pregnancy in case 21 was terminated based on the SNP array results. The fetus presented with bilateral VM and agenesis of the corpus callosum.

Prenatal diagnosis of recurrent 15q11.2 (BP1-BP2) microdeletion in a fetus presenting with VM, microcephaly, and fetal growth restriction was reported^36^. Recurrent BP1-BP2 microdeletion in 15q11.2 (including *TUBGCP5*, *CYFIP1*, *NIPA2*, and *NIPA1*) has been significantly associated with ID, DD, ASD, and behavioral and neurological problems^37^. The deletion of 15q11.2 is emerging as one of the most common SLs for NDDs. The deletion has been associated with two- to four-fold increase in prevalence—compared to control groups—of speech delay, autistic traits, and variable dysmorphism ^38^. For fetus 24, a 855 Kb deletion in 15q11.2 (BP1-BP2) was detected. The estimated penetrance of the variants was estimated to be 10.4%^33^. The pregnant couple elected to continue the pregnancy to term. The infant was followed-up for 2 years and 1 month. The infant presented with mild ID.

For CNVs with SL, considering the very limited observability of prenatal phenotypes, we propose that identification and follow-up of the carriers postnatally may explain the genetic etiology of NDDs. This proposal recognizes that environmental and/or other genetic factors may influence phenotypic expression of the SL^37^. There are no guidelines or consensus concerning the reporting of prenatally detected SLs, or concerning counseling. Some scholars have classified SL findings as pathogenic CNVs, although the penetrance is incomplete, and the phenotypes of control groups harboring SL were intermediate between the affected carrier groups and non-carrier control groups^39^. It is difficult to predict the risk of associated NDDs for a fetus with SL because ofthe lack of long-term follow-up data on such cohorts.

The diagnosis of an SL does not necessarily mean having to terminate the pregnancy. In cases of prenatal diagnosis of SL, the pregnancy outcome will depend on the severity of the fetal vital-organ sonographic anomalies detected. In our study, pregnancies were terminated based mainly on the severity of the fetal UA and the high penetrance of SL, especially occurring de novo. A high or moderate penetrance of an SL may prompt pregnancy termination for most couples. Our multidisciplinary discussion group considers that the timely diagnosis of SL can help the parents consider the symptoms associated with NDDs of their child as carefully as possible, and can further prompt active intervention by the parents as early as possible during childhood, especially for ASD. Only two such pregnancies (cases 17 and 24) in our cohort continued to term.

SNP array is utilized widely prenatally to detect submicroscopic anomalies and to characterize small supernumerary marker chromosomes (sSMCs) that cannot be characterized by G-banding. The prognosis of fetuses with sSMCs depends on the chromosomal origin and gene content. In our study, two fetuses (cases 19 and 29) with de novo sSMCs were diagnosed by G-banding. Furthermore, SNP array analysis revealed both fetuses carrying pathogenic CNVs associated with azoospermia and 21q11.2-q22.11 deletion syndrome, respectively. Both pregnancies were terminated.

SNP array also prenatally detects VOUS. The detection rate of VOUS by SNP array varies greatly^40^. In our study, six cases with VOUS were detected in groups I, II, and III. The detection rate of 2.7% was slightly higher than the 2.1% reported previously^41^. The difference may reflect the limited number of cases and cohort selection. Only two such pregnancies (cases 7 and 23) continued.

The diagnosis of VOUS poses challenges in genetic counseling and leads to parental anxiety. Parental SNP array verification, further functional study, long-term postnatal follow-up, large population study, investigation of family histories, and accumulated experience are vital for VOUS fetuses without an established family history.

Karyotyping and SNP array each have their respective advantages in chromosome analysis. Karyotyping is usefulfor detecting chromosomal balance recombination and mosaicism. SNP array is sensitive in identifying CNVs and LOH. The two technologies combined can provide more information for genetic counseling; they cannot be substituted for each other. In our study, SNP array failed to detect chromosome balanced chromosomal rearrangements (cases 28 and 31), and the detection rate of abnormal CNVs could be increased by an additional 7.2% via SNP array. Etiology contributing to 191 cases remained elusive, suggesting that environmental factors, single gene disease, epigenetics, and some syndromes may be involved in the etiology of VM. Therefore, next-generation sequencing (NGS) is recommended after viral infection and chromosome abnormalities are excluded.

Abnormal CNVs affect the obstetric outcomes. Among the 24 pregnant women harboring fetuses with pathogenic CNVs, 21 chose to terminate their pregnancy. Furthermore, we observed that only two pregnancies carrying VOUS elected to continue to term; pregnancies of four fetuses with VOUS (two fetuses presenting with fetal hydrocephalus and two fetuses with VM) were terminated.

Our study has some limitations. The sample size was small. Data are scant concerning the genetic etiology of VM. With the development and refinement of NGS, costs have decreased markedly; NGS technology outperforms microarray analysis and will broaden the exploration of the genome at single-nucleotide resolution to discover currently unknown aspects of the genome. Thus, the application of NGS, especially whole exome sequencing in fetal VM, should be pursued.

In conclusion, this retrospective study investigated the necessity to performing SNP array for VM fetuses with or without other UAs. SNP array has a higher detection rate of chromosomal abnormality than karyotyping in VM with or without other UAs; the CNVs detected will enrich the data concerning CNVs of fetal VM. SNP array may aid in the risk-assessment prognosis for fetuses with prenatal VM, especially in cases with CNS anomalies, regardless of other ultrasound results.

## Data Availability

The data that support the findings of this study are available from the corresponding author upon reasonable request.

## Abbreviations

CMA: Chromosomal microarray analysis
CNS: Central nervous system
CNV: Copy number variation
DD: Developmental delay
GDD: Global developmental delay
ID: Intellectual disability
IMVM: Isolated mild ventriculomegaly
IVM: Isolated ventriculomegaly
MVM: Mild ventriculomegaly
NDD: Neurodevelopmental disorder
NGS: Next-generation sequencing
NIVM: Non isolated ventriculomegaly
SNP: Single nucleotide polymorphism
SS: Sotos syndrome
sSMC: Small supernumerary marker chromosome
SVM: Severe ventriculomegaly
UA: Ultrasound anomaly
VM: Ventriculomegaly
VOUS: Variation(s) of unknown significance
WS: Wisconsin syndrome
